# Inclusion of bimetallic Fe_0.75_Cu_0.25_-BDC MOFs into Alginate-MoO_3_/GO as a novel nanohybrid for adsorptive removal of hexavalent chromium from water

**DOI:** 10.1038/s41598-022-23508-y

**Published:** 2022-11-09

**Authors:** Mohamed E. Mahmoud, Mohamed F. Amira, Mayar M. H. M. Azab, Amir M. Abdelfattah

**Affiliations:** Faculty of Sciences, Chemistry Department, Alexandria University, Moharem Bey, Alexandria, Egypt

**Keywords:** Materials science, Nanoscience and technology

## Abstract

Metal–organic frameworks (MOFs) as porous materials have recently attracted research works in removal of toxic pollutants from water. Cr(VI) is well-known as one of the most toxic forms of chromium and the selection of efficient and effective Cr(VI)-remediation technology must be focused on a number of important parameters. Therefore, the objective of this work is to fabricate a novel nanohybrid adsorbent for removal of Cr(VI) by using assembled bimetallic MOFs (Fe_0.75_Cu_0.25_-BDC)-bound- Alginate-MoO_3_/Graphene oxide (Alg-MoO_3_/GO) via simple solvothermal process. The aimed Fe_0.75_Cu_0.25_-BDC@Alg-MoO_3_/GO nanohybrid was confirmed by FTIR, SEM, TEM, XRD and TGA. Adsorptive extraction of Cr(VI) from aqueous solution was aimed by various optimized experimental parameters providing optimum pH = 3, dosage = 5–10 mg, starting concentration of Cr(VI) = 5–15 mg L^−1^, shaking time = 5–10 min. The point of zero charge (pH_Pzc_) was 3.8. For Cr(VI) removal by Fe_0.75_Cu_0.25_-BDC@Alg-MoO_3_/GO, four isotherm models were estimated: Langmuir, Freundlich, Temkin and Dubinin-Radushkevich (D-R) with calculated correlation coefficient (R^2^ = 0.9934) for Langmuir model which was higher than others. The collected results from the kinetic study clarified that *pseudo*-second order model is the most convenient one for describing the adsorption behavior of Cr(VI) and therefore, the adsorption process was suggested to rely on a chemisorption mechanism. Thermodynamic parameters referred that the adsorption mechanism is based on a spontaneous and exothermic process. Finally, the emerged Fe_0.75_Cu_0.25_-BDC@Alg-MoO_3_/GO nanohybrid was confirmed as an effective adsorbent for extraction of hexavalent chromium from real water specimens (tap, sea water and wastewater) with percentage recovery values > 98%.

## Introduction

The disposal of toxic materials from water has shown a great challenge for scientists to save the environment and human health from these hazardous substances. The discharged undesirable substances, in the aquatic environment are categorized as either organic contaminants viz. dyes, pesticides and antibiotics or inorganic contaminants as toxic heavy metals^1^. Heavy metals are toxic species that threaten humanity especially due to their impacts as carcinogenic and nonbiodegradable therefore, their pollution control has become one of the most concerns worldwide^2^. Industrial activities are representing the major source of pollution with toxic heavy metals including painting, mining, electroplating and others^3^. Hexavalent chromium (Cr (VI)) is an example of highly mobile, toxic, and non-biodegradable heavy metal ion with acceptable concentration limit in drinking water of 0.05 mg L^−1^ according to the World Health Organization (WHO)^4^.

Effective remediation methods of water containing toxic metal ions as Cr(VI) are needed and up till now, there are several investigated and reported methodologies as coagulation, photocatalytic reduction, membrane filtration, biodegradation and adsorption^5,6^. The adsorption method is highly recommended owing to its effectiveness, simplicity, and availability of a wide range of adsorbent materials^7^. Nowadays, a great number of materials were identified to exhibit good affinity to Cr(VI) and thus were effectively applied to eliminate it from water including metal oxides^8^, biochars^9^ and carbonaceous nanoparticles^10^**.** One of the drawbacks of the adsorption method is the long time that is needed to reach the equilibrium conditions and obtain the maximum recovery percentage^11^. Consequently, the synthesis of new classes of adsorbents with large surface area is generally aimed and required to save time and energy^12^.

Metal–organic Frameworks (MOFs) are excellent porous network structure materials that consist of metal ion and bridging organic ligands (connectors)^1^. These materials have been recently applied in in various fields as water remediation, drug delivery, gas storage and catalysis owing to their large specific surface area, ordered structure, facile modification, tunable porosity between microporous and mesoporous scale, possibility of preparing different interesting structures and the presence of various functional groups that are helpful for selective adsorption^13,14^. Based on such outlined promising features, MOFs have been reported as favorable adsorbents in the removal of contaminants from water. For example, Cu-based MOF was used in the removal of organic (methylene blue and levofloxacin drug) and inorganic (Cr (VI)) contaminants from water^1,15^. The adsorption and the catalytic performance of MOFs were also found to improve by changing organic ligands, grafting more active groups, introducing additional metal centers to pristine MOFs, and making composites with other convenient materials^16^**.** One of the most appropriate materials to prepare MOFs composites is carbon-based substances as graphene oxide (GO)^17^. Graphene oxide possesses numerous functional oxygen-containing groups with a two-dimensional structure by which graphene oxide can be introduced as a substrate for MOFs^18^. The structure of pure MOFs has free voids and spaces. Because of weak dispersion forces between their nanocrystals and based on their combination with GO fewer inter-void MOFs are obtained^19^**.** Also, GO increases the stability of MOFs in water via binding between functional groups of GO with metal ions in the MOFs structure and therefore, enhances the dispersive force with the MOFs by prevention of their aggregation and improving removal efficiency^20^. Moreover, the ability of graphene oxide to control the morphology and size of MOFs during the synthesis was also investigated and reported for removal of organic pollutants by GO-MOFs composites^21,22^.

Sodium alginate (Alg) is a natural biologically polysaccharide polymer that contains active functional groups (-OH and -COOH) and it has β–d-M (mannuronic acid) and α–l-G (guluronic acid)^23^. Alg is known to exhibit strong binding capabilities, renewability, and hydrophilic properties, so it is regarded as an excellent adsorbent for extraction of heavy metals and other pollutants from water^24^. Furthermore, nanometal oxides have been known for their effectiveness in water treatment, photocatalysis and supercapacitors owing to their high thermal and mechanical stability^25^. Herein, the nanometal oxide used is molybdenum trioxide (nano MoO_3_) due to its physiochemical properties and thermodynamic stability and its effectiveness in the removal of water pollutants based on the previous research^26^. The stability of molybdenum-based materials can be improved by its dispersion onto carbon support material as graphene oxide^27^.

The presence of multi-functional groups in bimetallic MOFs as well as the related unique properties of some oxides as GO and MoO_3_ have directed our attention to combine these three materials with sodium alginate as a biodegradable material, for the first time, to fabricate a novel chemical network structure of Fe_0.75_Cu_0.25_-BDC@Alg-MoO_3_/GO as a novel nanocomposite. The assembled nanocomposite characterized by the presence of reactive multifunctional groups with strong affinity and capability for binding and extraction of the toxic Cr(VI) from matrices^28^. In addition, the assembled Fe_0.75_Cu_0.25_-BDC@Alg-MoO_3_/GO nanocomposite was prepared by a green chemical method via simple solvothermal process and microwave-assisted synthesis technique. The applicability of the assembled nanocomposite was aimed to investigate and evaluate its performance in removal of Cr(VI), as an example of highly toxic heavy metal, from aqueous solution under the influence and impact of various experimental parameters as initial pH, shaking time, dosage, concentration, temperature and ionic strength.

## Materials and analytical methods

### Chemicals and instrumentations

The specifications of all chemicals and instruments are listed and compiled in Table 1 The employed chemicals were all of analytical grades and used without further purifications.

**Table 1:** Chemicals and instrumentations.

Chemicals	Chemical formula	Company	Molar mass (g/mol)	Assay
Graphite powder	C	NICE CHEMICALS	12.01	Particle size (150mesh): 99.5%
Potassium permanganate	KMnO_4_	BDH Chemicals Ltd	158.03	≥ 99.0%,
Terephthalic acid (H_2_BDC)	C_8_H_6_O_4_	Merck	166.13	≥ 98.0%
N,N Dimethylformamide (DMF)	C_3_H_7_NO	SDFCL fine-chem	73.09	99.0%
Iron(III) chloride hexahydrate	FeCl_3_.6H_2_O	Alpha chemika	270.3	99.0%
Copper nitrate trihydrate	Cu(NO_3_)_2_.3H_2_O	Merck	241.60	99.5%
Sodium alginate	C_6_H_7_O_6_Na	Alfa Aesar, UK	216.12	≥ 99.0%
Molybdenum trioxide	MoO_3_	VEB laborchemie Apolda	143.94	99.5%
Glutaldehyde	C_5_H_8_O_2_	BDH chemicals Ltd. England	100.11	> 95.0%
1,5-Diphenylcarbazide	C_13_H_14_N_4_O	Sigma-Aldrich	242.28	≥ 99.0%
Sulfuric acid	H_2_SO_4_	Sigma-Aldrich	98.07	98.0%
Sodium hydroxide	NaOH	Riedel de Haen, Germany	40.00	≥ 99.0%
Hydrochloric acid (HCl)	HCl	Sigma Aldrich,USA	36.46	37.0% (w/w)
Sodium chloride	NaCl	VWR international Ltd	58.44	99.5%

Measurements	The model of the instrument			
UV–Vis spectrophotometer	Unico UV– Vis-7200			
pH values	Adwa pH-meter			
microwave oven	KOG-1B5H, Korea, 1400 W and 2.45 GHZ			
Fourier transform infrared (FT-IR)	BRUKER Tensor 70			
Scanning electron microscope (SEM)	JEOL-JSM-5300			
High Resolution Transmission Electron Microscope (HR-TEM)	JEOL- JSM			
X-ray diffraction (XRD)	JED-2300 T model			
Thermal gravimetric analysis (TGA)	Linseis STA PT1000			

### Experimental

#### *Microwave synthesis of Alg-MoO*_*3*_*/GO*

GO was first prepared by oxidation of graphite powder by KMnO_4_ in the presence of sulfuric acid. In a brief, 5 g of graphite were mixed with 15 mL of concentrated H_2_SO_4_ and 25 mL of 0.5 M KMnO_4_, and then the mixture was heated in the microwave oven for 5 min at 80 °C allowing the oxidation process to proceed. The step of adding KMnO_4_ was repeated 4 times to the point of black precipitation of GO. This was collected and washed with DW till neutrality and finally, it was dried at the oven (65 °C)^29^.

Alg-MoO_3_ was synthesized by adding 20 mL of DW in a mixture containing 2 g sodium alginate and 2 g molybdenum trioxide. This mixture was exposed to microwave irradiation for 5 min at 80 °C. The step of adding DW was repeated 4 times to ensure the complete binding process of Alg with MoO_3_^30^.

Alg/GO was synthesized by adding 20 mL of DW in a mixture of sodium alginate and GO with a ratio 1:1 (w/w). This mixture was heated by microwave irradiation for 5 min and the step of adding DW was repeated 4 times to ensure the complete binding process of Alg with GO^31^.

Synthesis of Alg-MoO_3_/GO was accomplished by mixing 20 mL of glutaraldehyde as a crosslinker with a mixture of Alg-MoO_3_ and Alg/GO in a 1:1 (w/w) ratio. This resultant mixture was then exposed and heated by microwave irradiation at 80 oC for 5 min and the obtained black precipitate of Alg-MoO_3_/GO was collected and dried at 65 °C in an oven overnight^30,31^.

#### *Synthesis of bimetallic MOFs (Fe*_*0.75*_*Cu*_*0.25*_*-BDC)*

The bimetallic MOFs (Fe_0.75_Cu_0.25_-BDC) were prepared by using a solvothermal method. In this process, FeCl_3_.6H_2_O (7.5 mmol) was added to Cu(NO_3_)_2_.3H_2_O (2.5 mmol) in 30 mL DMF. This was then poured into a solution of H_2_BDC (5 mmol) that had already been dissolved in 30 mL DMF and exposed to magnetic stirring for 30 min at room temperature to form a clear solution. The above mixture was placed into a Teflon-lined autoclave with a volume of 100 mL and allowed to react for 36 h at 120 °C. After completion of the reaction, the obtained MOFs precipitate (Fe_0.75_Cu_0.25_-BDC) was washed with DMF and DW and then dried in a 70 °C oven overnight^32^.

#### *Synthesis of Fe*_*0.75*_*Cu*_*0.25*_*-BDC@Alg-MoO*_*3*_*/GO nanohybrid*

**Figure 1:**
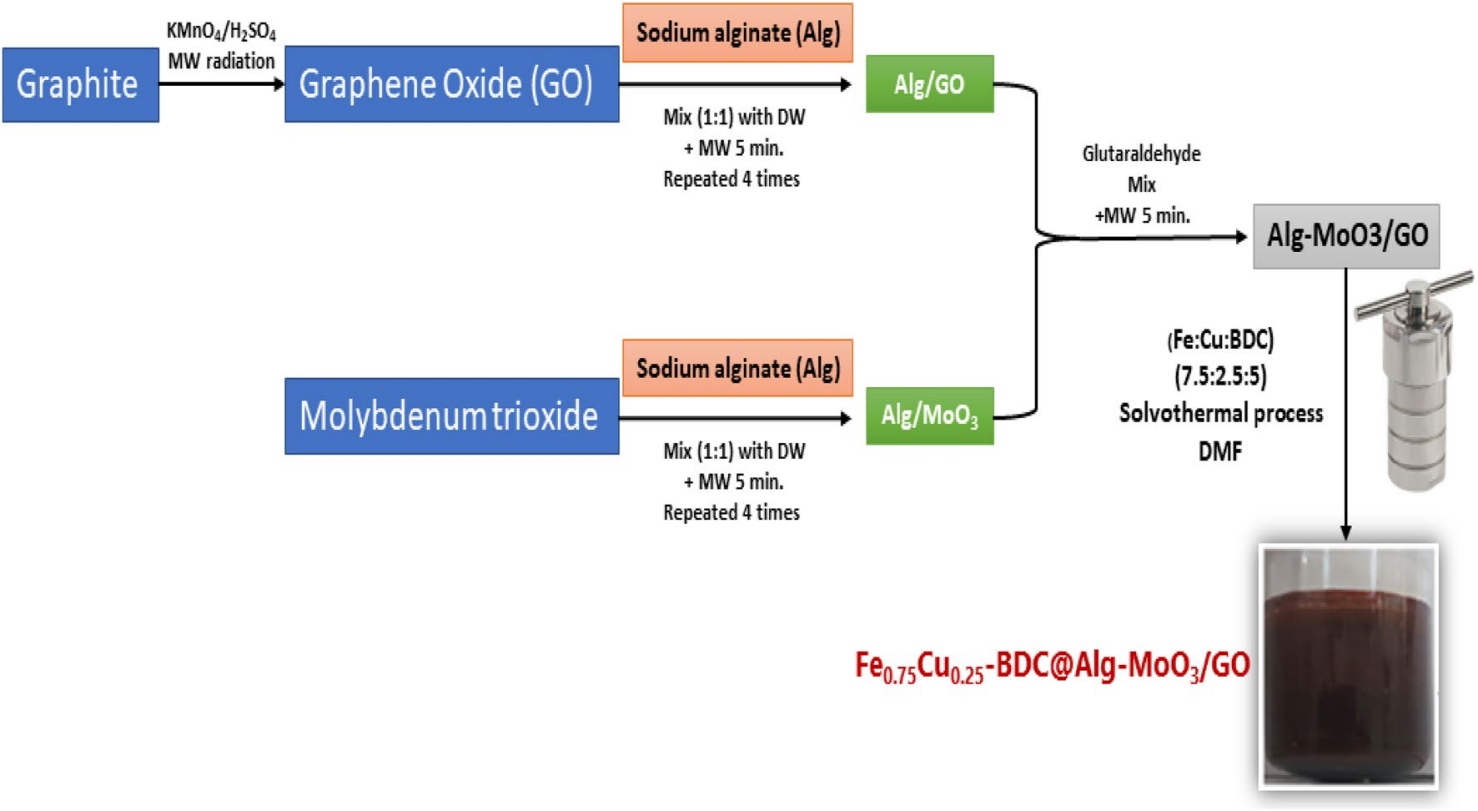
Preparation of Fe_0.75_Cu_0.25_-BDC@Alg-MoO_3_/GO.

Fe_0.75_Cu_0.25_-BDC was introduced to Alg-MoO_3_/GO by a simple solvothermal process. The dried Alg-MoO_3_/GO was used as a platform for the growth of Fe_0.75_Cu_0.25_-BDC MOFs on its surface. Typically, 0.25 g of Alg-MoO_3_/GO in 20 mL DMF was stirred for 1 h, then 1 g of H_2_BDC was well dissolved in 35 mL DMF and poured into Alg-MoO_3_/GO for 30 min stirring. The suspension was then stirred for 1 h at room temperature with dissolved 2.5 g FeCl_3_.6H_2_O and 0.74 g Cu(NO_3_)_2_.3H_2_O in 30 mL DMF. Finally, the attained mixture was placed into a Teflon-lined autoclave with a volume of 100 mL and allowed to react for 36 h at 120 °C. The Fe_0.75_Cu_0.25_-BDC@Alg-MoO_3_/GO nanohybrid was separated by filtration then washed with DMF and DW and dried in a 65 °C oven. Finally, a flowchart diagram is provided to illustrate all preparation steps and procedures of Fe_0.75_Cu_0.25_-BDC@Alg-MoO_3_/GO nanohybrid as shown in Fig. 1.

### Adsorption behavior of Fe_0.75_Cu_0.25_-BDC@Alg-MoO_3_/GO nanohybrid

The adsorption behavior of hexavalent chromium ion onto Fe_0.75_Cu_0.25_-BDC@Alg-MoO_3_/GO nanohybrid was optimized by the batch operation at different factors. The source of Cr(VI) ion was potassium dichromate and the required concentration (5, 10 and 15 mg L^−1^) was prepared via dilution of the stock solution (1000 mg L^−1^). The maximum wavelength of Cr(VI) is 540 nm that was detected in presence of 1,5 diphenylcarbazide. In this study, Fe_0.75_Cu_0.25_-BDC@Alg-MoO_3_/GO nanohybrid was added to 10 mL of hexavalent chromium ion solution. After 30 min of shaking time, the mixture was filtered and the concentration of filtrate was estimated by spectrophotometric determination at λ_max_ 540 nm. The removal percentage was evaluated from Eq. (1).

1 $$\% ~E = \frac{{C_{{\text{o}}} - C}}{{C_{{\text{o}}} }} \times 100$$

The initial and residual concentrations of chromium ion is represented by C_o_ and C (mol L^−1^) respectively.

Firstly, the influence of varying pH 2–7 was studied for three concentrations of chromium ion solution (5, 10 and 15 mg L^−1^). The values of pH were monitored utilizing diluted drops of either HCl or NaOH (0.1 mol L^−1^). 20.0 mg of Fe_0.75_Cu_0.25_-BDC@Alg-MoO_3_/GO nanohybrid was mixed with 10 mL of the selected concentration of Cr(VI) ion solution then a batch operation was allowed for 30 min. The mixture was filtered and the residual chromium ion in the filtrate was estimated at λ_max_ 540 nm. Finally, the removal percentages (%E) were computed from Eq. (1).

Furthermore, the point of zero charge (PZC) of Fe_0.75_Cu_0.25_-BDC@Alg-MoO_3_/GO nanohybrid was performed by addition of 0.1 mol L^−1^ sodium chloride with definite volume to 100 mg of the prepared nanohybrid, then the pH was adjusted from 2 to 11, while the batch operation was applied for 4 h. The final pH was estimated after 24 h.

The influence of reaction time on removal of Cr(VI) ion by Fe_0.75_Cu_0.25_-BDC@Alg-MoO_3_/GO nanohybrid was studied using time intervals 5–80 min. 20.0 mg of nanohybrid was mixed with 10 mL of the selected concentration of chromium ion (5, 10 and 15 mg L^−1^) at pH 3 (optimum pH) at room temperature. From the data of this factor, the kinetic models were investigated to elucidate the extraction mechanism(s) of Cr(VI) by Fe_0.75_Cu_0.25_-BDC@Alg-MoO_3_/GO nanohybrid.

The dosage influence of Fe_0.75_Cu_0.25_-BDC@Alg-MoO_3_/GO nanohybrid on the removal of hexavalent chromium ion was also investigated utilizing different masses 5–70 mg. The chosen mass was mixed with 10 mL of the selected concentration of chromium ion (5, 10 and 15 mg L^−1^) at pH 3 for 30 min.

The influence of the initial concentration of the solution of chromium ion was studied with various concentrations from 5 to 100 mg L^−1^ in which 20.0 mg of Fe_0.75_Cu_0.25_-BDC@Alg-MoO_3_/GO nanohybrid was mixed with 10 mL of chromium solution and each prepared concentration was adjusted at pH 3. The removal percentage was estimated from Eq. (1). The data collected from this factor were used to investigate the isotherm study and to understand the mechanism of adsorption of the contaminant.

The influence of temperature on the extraction of Cr(VI) was also studied utilizing distinct values of temperature (298, 307, 316, 323 and 328 K). 20.0 mg of Fe_0.75_Cu_0.25_-BDC@Alg-MoO_3_/GO nanohybrid was added to 10 mL of the selected concentration of chromium solution (5, 10 and 15 mg L^−1^) at pH 3. This factor was also employed to study the thermodynamics parameters of Cr(VI) ion removal onto Fe_0.75_Cu_0.25_-BDC@Alg-MoO_3_/GO nanohybrid.

The influence of ionic strength using various masses (5–100 mg) of sodium chloride electrolyte was also studied. The chosen mass of the electrolyte was mixed with 20.0 mg of Fe_0.75_Cu_0.25_-BDC@Alg-MoO_3_/GO nanohybrid in 10 mL of Cr(VI) ion solution (5, 10 and 15 mg L^−1^) that was adjusted at pH 3. The Cr(VI) ion removal percentage was estimated as before from Eq. (1).

The recycling capability of Fe_0.75_Cu_0.25_-BDC@Alg-MoO_3_/GO was also investigated by applying 0.05 mol L^−1^ HCl as the regeneration reagent to activate the loaded Fe_0.75_Cu_0.25_-BDC@Alg-MoO_3_/GO nanohybrid with hexavalent chromium ion. 100 mg Fe_0.75_Cu_0.25_-BDC@Alg-MoO_3_/GO nanohybrid was mixed with 10 mL of hexavalent chromium solution at pH 3 and shaken for 30 min. The loaded nanohybrid was then washed with HCl and DW and dried to be ready for the recycling operation.

Finally, the potential applicability of Fe_0.75_Cu_0.25_-BDC@Alg-MoO_3_/GO nanohybrid for removal of hexavalent chromium ion from real water specimens (tap, seawater and industrial wastewater) was also investigated in this study. This study was carried out at optimum conditions using three concentrations of spiked Cr(VI) ion solution (5, 10 and 15 mg L^−1^). 70.0 mg of Fe_0.75_Cu_0.25_-BDC@Alg-MoO_3_/GO was mixed with 10 mL of spiked solution at pH 3. Water specimens used in this factor were drinking water from the tap, seawater from the Mediterranean Sea and eventually collected wastewater from Elmahmodya canal, Alexandria, Egypt.

## Results and discussions

### Characterization

#### Fourier transform- infrared (FT-IR) study

**Figure 2:**
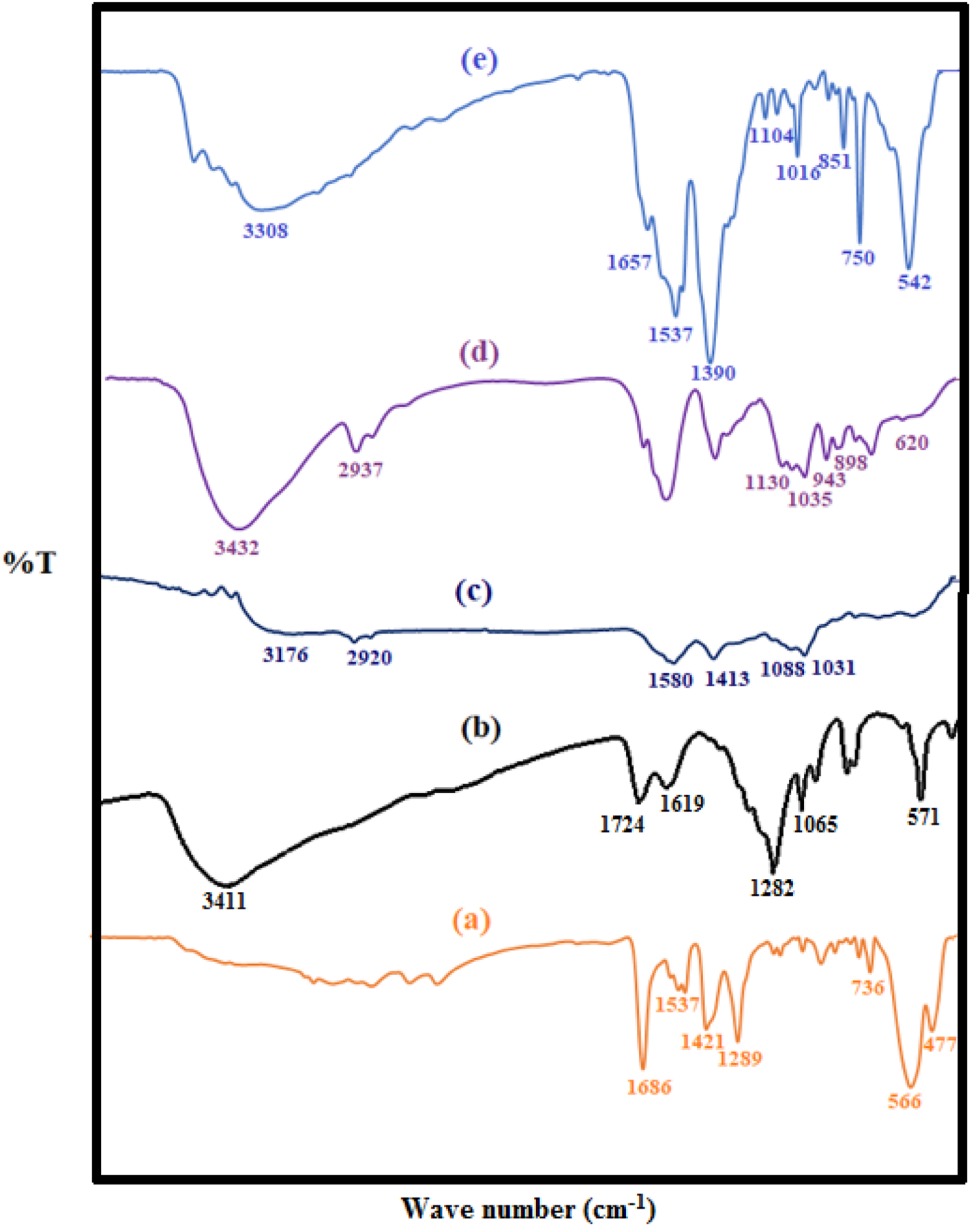
FT-IR spectra of (**a**) Fe_0.75_Cu_0.25_-BDC MOFs, (**b**) GO, (**c**) Alg-GO, (**d**) Alg-MoO_3_/GO and (**e**) Fe_0.75_Cu_0.25_-BDC@Alg-MoO_3_/GO.

The FT-IR spectra of Fe_0.75_Cu_0.25_-BDC MOFs, GO, Alg-GO, Alg-MoO_3_/GO and Fe_0.75_Cu_0.25_-BDC@Alg-MoO_3_/GO were acquired to confirm their structures. The chemical structure of Fe_0.75_Cu_0.25_-BDC MOFs is represented by the illustrated FTIR spectrum in Fig. 2. There is no broad peak found at 3400 cm^−1^ which proves the absence of O–H stretching vibration. The characteristic absorption bands at 1686, 1537, 1421 and 1289 cm^−1^ are assigned to the vibration of the carboxylate groups which coordinated with Cu^2+^ and Fe^3+^ in Fe_0.75_Cu_0.25_-BDC MOFs^33^. The sharp band that appeared at 736 cm^−1^ is attributed to the C–H bending vibration of the benzene ring, while the band at 566 cm^−1^ refers to the Cu–O stretching vibration^34^, while, the absorption band displays at 477 cm^−1^ is due to the Fe–O stretching vibration^35^. The result of FT-IR analysis confirms the presence of a metal-oxo bond between the C = O of terephthalic acid and metal ions (Cu^2+^ and Fe^3+^) to refer to the successful formation of bimetallic Fe_0.75_Cu_0.25_-BDC MOFs. The FT-IR spectrum of microwave-synthesized graphene oxide was also studied. The presence of a broad band at 3411 cm^-1^ is assigned to the stretching vibrations of the O–H bond to prove the successful oxidation of graphite ^36^. The peak at 1724 cm^-1^ is attributed to the C = O stretching vibrations of the carboxylic acid in GO. There are also some C = C bonds of sp^2^ carbons in the 1619 cm^−1^ region. The presence of a peak at 571 cm^−1^ in GO may be attributed to the presence of traces of Mn–O as the employed oxidizing agent^37,38^. The displayed peaks at 3176 cm^−1^ and 2920 cm^−1^ in the Alg-GO spectrum denote to the O–H and CH_2_ stretching vibrations from graphene oxide and/or alginate, while the bands at 1088 and 1031 cm^−1^ are attributed to glucuronic and mannuronic units, respectively^39^. The observed bands at 1580 and 1413 cm^−1^ are evident in the symmetric stretching frequency and the asymmetric one of the carboxylic acid group of alginates^40^. In the FT-IR spectrum of Alg-MoO_3_/GO, a broad peak was found at 3432 cm^−1^ that corresponds to the O–H stretching vibrations in GO and/or alginate, while the peak at 2937 cm^−1^ is assigned to C-H bond vibration. The characteristic peaks of MoO_3_ are identified at 944 and 620 cm^−1^ which are attributed to Mo = O bonds, Mo–O–Mo vibrations of Mo^6+^ and Mo–O–Mo, respectively. The peaks of epoxy group of graphene oxide are located at 1035 and 1130 cm^−1^ in Alg-MoO_3_/GO and therefore, the epoxy-functional groups may be attached to molybdenum trioxide^41^. The peak at 3308 cm^−1^ in Fe_0.75_Cu_0.25_-BDC@Alg-MoO_3_/GO, corresponds to stretching vibrations of O–H of GO. The peaks at 1657 cm^−1^,1537 cm^−1^ and 1390 cm^−1^ are evident to carbonyl group (COOH) asymmetric and symmetric vibrations and sp^2^ C = C bond. The peaks at 1104 cm^−1^ and 1016 cm^−1^ correspond to stretching vibrations of C–O bond in COO– and C–O–H. The peaks resulting in stretching vibrations of metal-O bonds are confirmed by their presence at 851 cm^−1^, 750 cm^−1^ and 542 cm^−1^ respectively.

#### Scanning and transmission electron microscopic analysis

**Figure 3:**
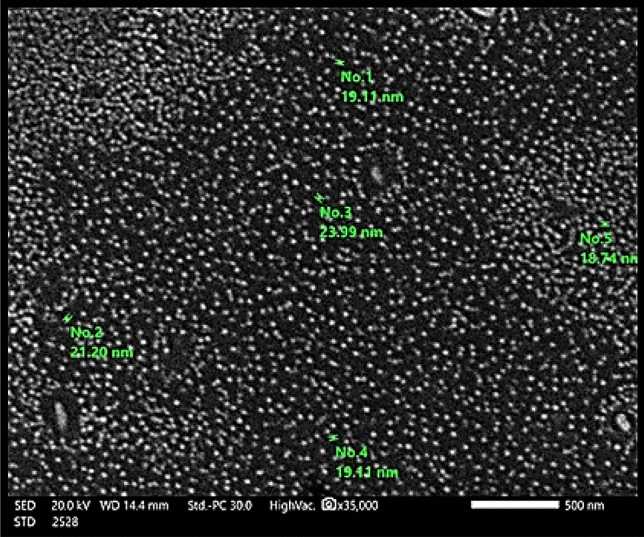
SEM image of Fe_0.75_Cu_0.25_-BDC@Alg-MoO_3_/GO.

**Figure 4:**
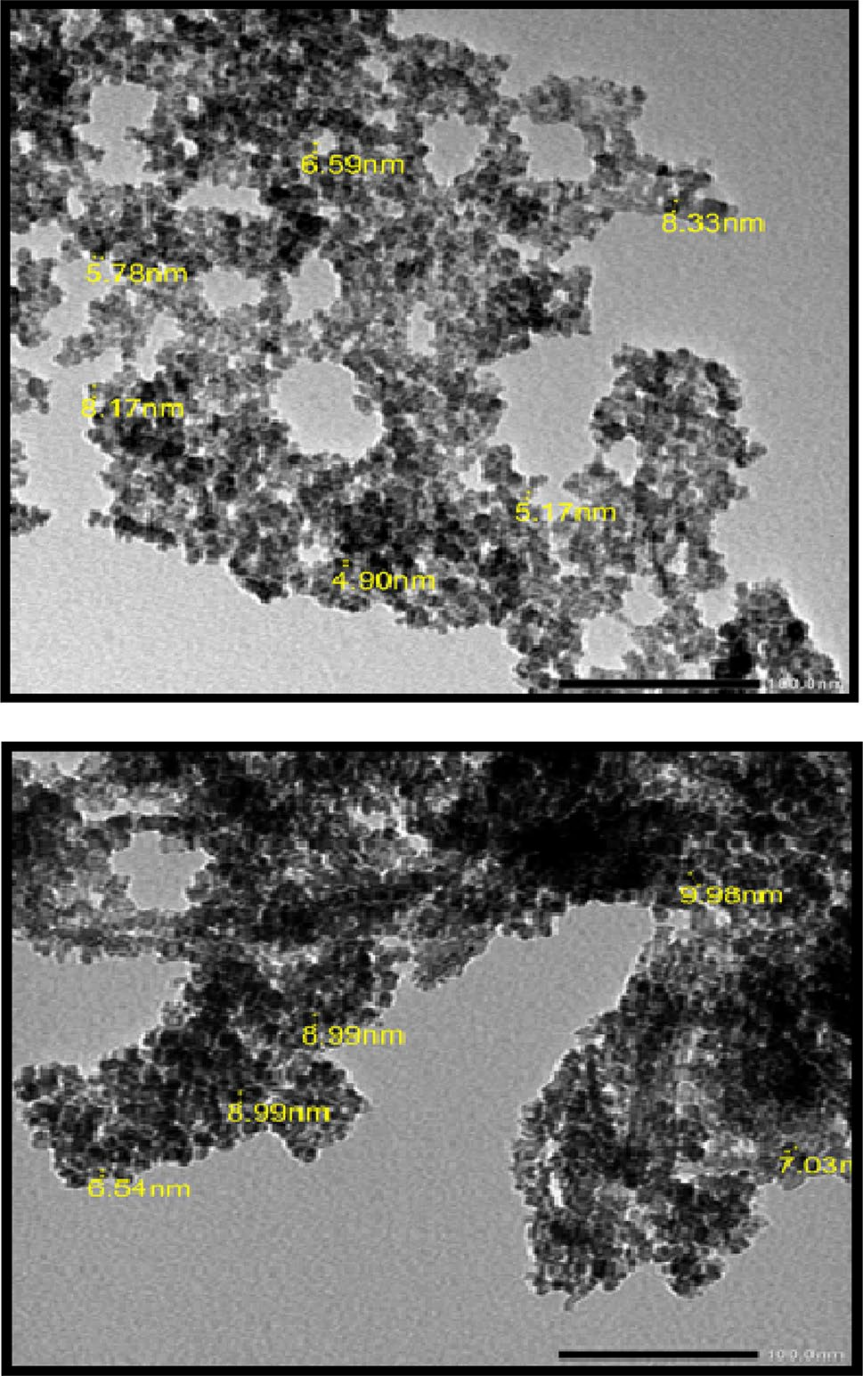
TEM image of Fe_0.75_Cu_0.25_-BDC@Alg-MoO_3_/GO.

SEM characterization refers to the surface morphology of Fe_0.75_Cu_0.25_-BDC@Alg-MoO_3_/GO at magnification order × 35.000 as illustrated in Fig. 3. The SEM image shows that the particles of Fe_0.75_Cu_0.25_-BDC@Alg-MoO_3_/GO nanohybrid are spherical in shape and homogenously distributed with no aggregation. Furthermore, the average particle size in the assembled Fe_0.75_Cu_0.25_-BDC@Alg-MoO_3_/GO nanohybrid material was characterized as 20.52 nm. This fact proves the successful preparation of Fe_0.75_Cu_0.25_-BDC@Alg-MoO_3_/GO at nanoscale range thus providing good adsorption of Cr(VI) ions. The morphology of Fe_0.75_Cu_0.25_-BDC@Alg-MoO_3_/GO nanohybrid was further proved by the TEM imaging Fig. 4. which elucidates that the prepared nanohybrid was spherical in shape with accurate particle size distribution in the range from 4.19 nm to 8.33 nm. The image also shows the good distribution of nanosized Fe_0.75_Cu_0.25_-BDC@Alg-MoO_3_/GO.

#### X-Ray diffraction

**Figure 5:**
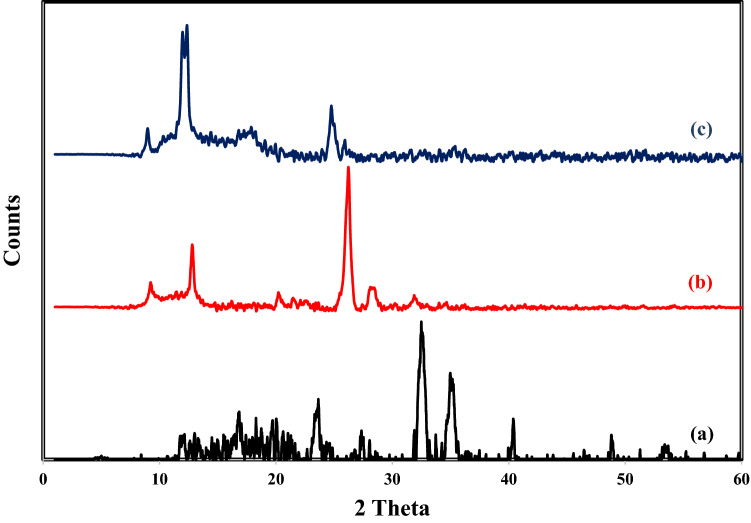
XRD patterns of (**a**) Fe_0.75_Cu_0.25_-BDC MOFs, (**b**) Alg-MoO_3_/GO and (**c**) Fe_0.75_Cu_0.25_-BDC@Alg-MoO_3_/GO.

XRD is a significant non-destructive method to determine the crystallinity of a material. Herein, the XRD pattern of Fe_0.75_Cu_0.25_-BDC MOFs, Alg-MoO_3_/GO and Fe_0.75_Cu_0.25_-BDC@Alg-MoO_3_/GO was performed as illustrated in Fig. 5. The XRD pattern of Fe_0.75_Cu_0.25_-BDC MOFs shows some characteristic peaks at 2θ = 12.11° 16.80° 20.06° 23.61° and 32.5° which prove the successful incorporation of copper in Fe_0.75_Cu_0.25_-BDC MOFs^42^. The XRD curve of Alg-MoO_3_/GO indicates the crystalline structure with a broad reflection around 11.62° as a characteristic peak for alginate^43^. Furthermore, the diffraction peaks at 2θ = 9.32°, 12.93° and 25.95° correspond to the graphene oxide and molybdenum trioxide which consistent with the standard graphite JCPDS file no. 75-2078 and ICDD sheet no. 00-021-0569 respectively^44–47^.

Finally, the XRD pattern of Fe_0.75_Cu_0.25_-BDC@Alg-MoO_3_/GO nanohybrid is quite different and shows several diffraction peaks at 2θ = 9.01° 12.38° 17.93° and 24.75° which clearly illustrate the increase in the crystallinity of the final nanohybrid as well as its successful preparation.

#### Thermal analysis

**Figure 6:**
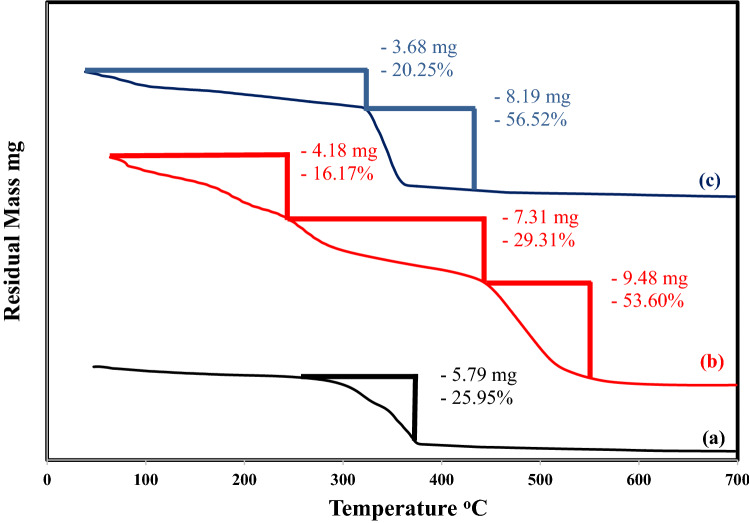
TGA thermograms (**a**) Fe_0.75_Cu_0.25_-BDC MOFs, (**b**) Alg-MoO_3_/GO and (**c**) Fe_0.75_Cu_0.25_-BDC@Alg-MoO_3_/GO.

The thermal stability Fe_0.75_Cu_0.25_-BDC MOFs, Alg-MoO_3_/GO and Fe_0.75_Cu_0.25_-BDC@Alg-MoO_3_/GO nanohybrid was studied by TGA analysis. The stages of weight loss are illustrated in the TGA diagram (Fig. 6). Firstly, Fe_0.75_Cu_0.25_-BDC MOFs exhibited only one degradation stage with 25.29% mass loss that could be due to the decomposition of volatile compounds and no more mass loss above 377 °C to refer to the good thermal stability of the prepared bimetallic MOF ^48^. There are three degradation stages of Alg-MoO_3_/GO. The first step is evident with a mass loss 16.17% due to the evaporation of water molecules adsorbed on the surface. The second step is evident with a mass loss of 29.31% as sodium alginate began to decompose and the degradation of oxygen-containing functional groups in GO, while the third one with 53.60% loss due to further cracking of sodium alginate and decomposition of GO into CO and CO_2_^30^. Finally, the thermogram of Fe_0.75_Cu_0.25_-BDC@Alg-MoO_3_/GO nanohybrid elucidates two decomposition steps with a total mass loss of 76.77% due to the above-mentioned degradation steps.

### Adsorption optimization

#### Impact of initial pH values

**Figure 7:**
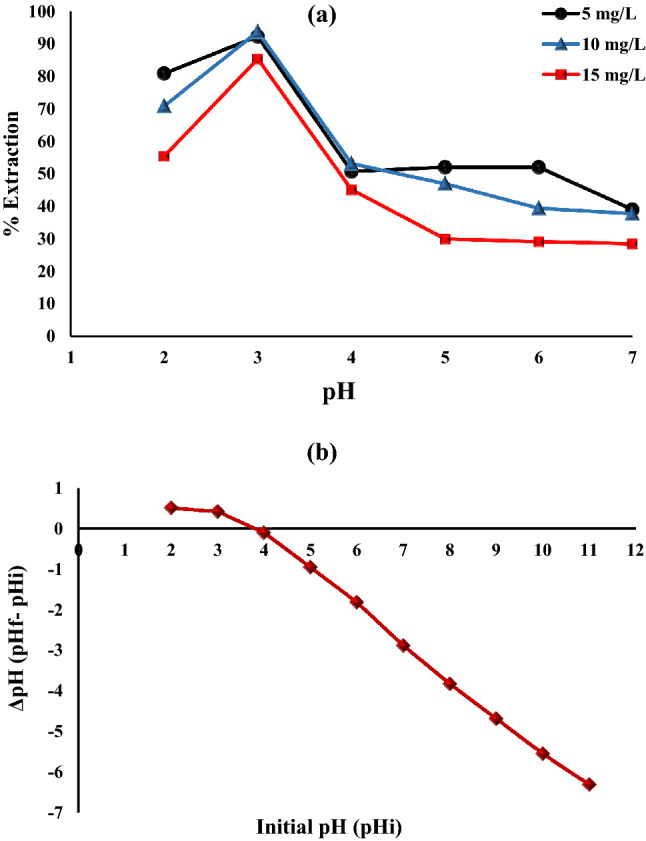
(**a**) Impact of initial pH on the removal of Cr(VI) by Fe_0.75_Cu_0.25_-BDC@Alg-MoO_3_/GO (**b**) PZC plot.

The initial pH of the solution is a significant factor as it affects the charge on the surface of Fe_0.75_Cu_0.25_-BDC@Alg-MoO_3_/GO nanohybrid and its ionized forms as well as metal species. Figure 7a. depicts the removal of Cr(VI) onto Fe_0.75_Cu_0.25_-BDC@Alg-MoO_3_/GO nanohybrid under pH range values from 2 to 7 using three different concentrations of Cr(VI) ions (5, 10 and 15 mg L^−1^). It’s evident that the removal percentages of the three chromium ion concentrations were decreased with increasing pH values. The optimum removal percentages were achieved at pH 3 as 92.27%, 93.27 and 85.32% by 5, 10 and 15 mg L^−1^ respectively. Hexavalent chromium is favorably adsorbed at low pH onto Fe_0.75_Cu_0.25_-BDC@Alg-MoO_3_/GO nanohybrid owing to the protonation of the functional groups at the surface of nanohybrid at low pHs. Thus, the binding and electrostatic attraction between the surface and anionic chromium at pH 3 dominated the adsorption process. Furthermore, the removal percentage values were decreased at higher pH values because of the repulsion force between the negatively charged surface of Fe_0.75_Cu_0.25_-BDC@Alg-MoO_3_/GO nanohybrid and anionic hexavalent chromium to favor less available active sites on the surface^49^.

The point of zero charge of the prepared Fe_0.75_Cu_0.25_-BDC@Alg-MoO_3_/GO nanohybrid was also studied for further confirmation of the effect of pH on the adsorption mechanism. The PZC is determined when the surface of the nanohybrid becomes neutral. Figure 7b represents the plot between ΔpH (pH_final_- pH_initial_) versus pH_initial_ from which the value of PZC was obtained at pH_Pzc_ = 3.8. At pH < pH_Pzc_ the surface is positively charged and therefore, it is favorable to adsorb anions by electrostatic attraction force^50^**.**

#### Impact of time and kinetic studies

**Figure 8:**
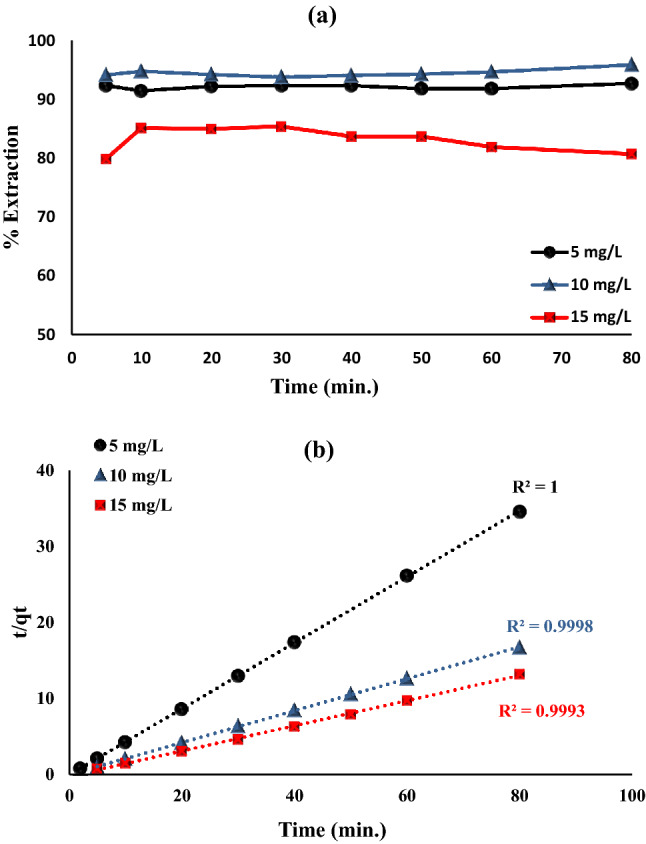
(**a**) Impact of time on Cr(VI) ions removal by Fe_0.75_Cu_0.25_-BDC@Alg-MoO_3_/GO and (**b**) *Pseudo*-second order model for removal of Cr(VI) ions.

The influence of time using batch operation between Fe_0.75_Cu_0.25_-BDC@Alg-MoO_3_/GO and hexavalent chromium ion is necessary for studying the mechanism(s) of adsorption. The time range was adjusted from 5 to 80 min utilizing 20 mg of Fe_0.75_Cu_0.25_-BDC@Alg-MoO_3_/GO nanohybrid with 10 mL of hexavalent chromium ion (5, 10 and 15 mg L^−1^) at room temperature and pH 3 and the collected results are shown in Fig. 8a. It’s obvious that the removal of Cr(VI) almost remained constant as the shaking time increases. The removal percentages reached equilibrium after only five minutes for 5 mg L^−1^ and 10 mg L^−1^, while 10 min for 15 mg L^−1^ concentrations. The characterized maximum removal percentages were 92.28%, 94.08% and 85.07% for 5, 10 and 15 mg L^−1^ respectively. This proves the efficient binding between the active sites of Fe_0.75_Cu_0.25_-BDC@Alg-MoO_3_/GO nanohybrid with Cr(VI) as it reached full saturation very fast^51^.

Kinetic study is very significant as it elucidates the rate of adsorption of adsorbate at solid/liquid interface. The mechanism of adsorption of hexavalent chromium ion onto Fe_0.75_Cu_0.25_-BDC@Alg-MoO_3_/GO nanohybrid was studied by using four various kinetic techniques; *pseudo*-first order, *pseudo*-second order, intra-particle diffusion and Elovich kinetic models. The first studied model for adsorption of Cr(VI) onto Fe_0.75_Cu_0.25_-BDC@Alg-MoO_3_/GO nanohybrid is the *pseudo*-first order and its related parameters are computed from Eq. (2).

2 $$\ln \left( {{\text{q}}_{e} - {\text{q}}_{{\text{t}}} } \right) = \ln {\text{q}}_{e} - {\text{k}}_{1} {\text{t}}$$

where the adsorption capacities at equilibrium and at any time t can be denoted by ${\text{q}}_{e}$ and ${\mathrm{q}}_{\mathrm{t}}$ respectively. The ${\mathrm{k}}_{1}$ describes the value of rate constant (min^−1^). The R^2^ values for *pseudo*-first order model were very low (R^2^ = 0.166, 0.3770 and 0.1727). Therefore, this model is inappropriate to describe this adsorption mechanism of hexavalent chromium ion onto Fe_0.75_Cu_0.25_-BDC@Alg-MoO_3_/GO nanohybrid. Similarly, the *pseudo*-second order kinetic parameters are calculated from Eq. (3).

3 $$\frac{{\text{t}}}{{{\text{q}}_{{\text{t}}} }} = \frac{1}{{{\text{k}}_{2} {\text{q}}_{{\text{e}}}^{2} }} + \frac{{\text{t}}}{{{\text{q}}_{{\text{e}}} }}$$

where, the rate constant for *pseudo*-second order is denoted by ${\mathrm{k}}_{2}$ (g/mg.min). This model was examined by plotting t/${\mathrm{q}}_{\mathrm{t}}$ vs t (Fig. 8b). The correlation coefficients for this model were 1.000, 0.9998 and 0.9993 for 5, 10 and 15 mg L^−1^ chromium ion concentrations, respectively. Furthermore, the q_e(calc)_ was nearly equal to q_e(exp)_. So, the adsorption behavior obviously fit this model to illustrate that the adsorption reaction of hexavalent chromium ion via multi active sites on the nanohybrid surface. The intra-particle diffusion and Elovich models were both studied from equations Eqs. (4) and (5), respectively. It was found that these two models were inconvenient to elucidate adsorption behavior of Cr(VI) onto Fe_0.75_Cu_0.25_-BDC@Alg-MoO_3_/GO nanohybrid based on the computed R^2^ values^52^.

4 $${\mathrm{q}}_{\mathrm{t}}={\mathrm{k}}_{\mathrm{id}}{\mathrm{t}}^\frac{1}{2}+\mathrm{C}$$

5 $${\mathrm{q}}_{\mathrm{t}}=\frac{1}{\upbeta }\mathrm{ln\alpha \beta }+\frac{1}{\upbeta }\mathrm{lnt}$$

Finally, the collected results from the kinetic study clarify that *pseudo*-second order model is the most convenient one to depict the adsorption behavior of hexavalent chromium ion onto Fe_0.75_Cu_0.25_-BDC@Alg-MoO_3_/GO nanohybrid and therefore, the adsorption behavior is suggested to rely on a chemisorption mechanism (chemically rate control mechanism)^53^.

#### *Impact of Fe*_*0.75*_*Cu*_*0.25*_*-BDC@Alg-MoO*_*3*_*/GO dosage*

**Figure 9:**
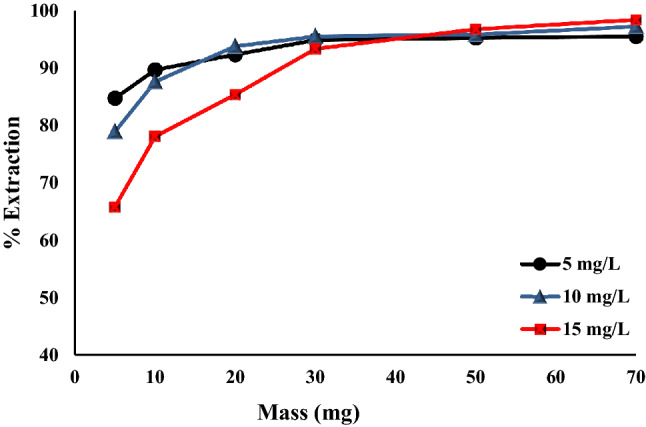
The dosage impact of Fe_0.75_Cu_0.25_-BDC@Alg-MoO_3_/GO.

The selected mass of Fe_0.75_Cu_0.25_-BDC@Alg-MoO_3_/GO nanohybrid is known to affect the uptake of various adsorbates as Cr(VI). Figure 9 depicts the relationship between Fe_0.75_Cu_0.25_-BDC@Alg-MoO_3_/GO dosages (5–70 mg) with the Cr (VI) removal percentages by using three concentrations of hexavalent chromium ion (5, 10 and 15 mg L^−1^). It can be outlined that the removal percentages of hexavalent chromium were initially increased with the increase of Fe_0.75_Cu_0.25_-BDC@Alg-MoO_3_/GO dosage and then reached to the maximum value at 30 mg. The removal percentages increased from 84.68%, 78.86% and 65.77% to 94.81%, 95.45% and 93.28% by using 5, 10 and 15 mg L^−1^, respectively. This may be attribute to the structure of Fe_0.75_Cu_0.25_-BDC@Alg-MoO_3_/GO nanohybrid which provides a great number of functional groups at the surface to enable efficient adsorption of the contaminant^54^.

#### Impact of initial Cr(VI) concentration and adsorption isotherms

**Figure 10:**
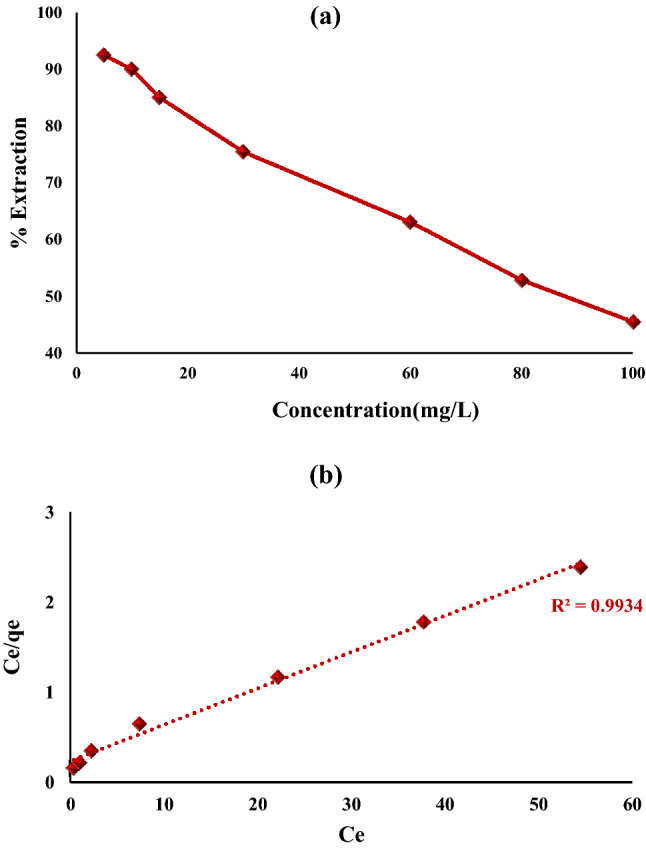
(**a**) Impact of initial concentration on Cr(IV) ions removal by Fe_0.75_Cu_0.25_-BDC@Alg-MoO_3_/GO and (**b**) Langmuir model.

Various concentrations of hexavalent chromium are known to affect its removal and uptake values by Fe_0.75_Cu_0.25_-BDC@Alg-MoO_3_/GO nanohybrid. It has been confirmed that the removal percentages was declined from 92.41% to 45.56% when the initial concentration was raised from 5 to 100 mg L^−1^ as illustrated in Fig. 10a. This declining order is due to the decrease in the number of nanohybrid particles compared to Cr(VI) ratio. However, as the concentration of Cr(VI) increased, while maintaining the active sites of Fe_0.75_Cu_0.25_-BDC@Alg-MoO_3_/GO nanohybrid constant, a great number of chromium ion were left unadsorbed. So, the deficiency of active sites at higher concentrations of the contaminant led to a decrease in the removal efficiency for Cr(VI)^55^.

Herein, four adsorption isotherm models (Langmuir, Freundlich, Dubinin-Radushkevich and Temkin) were studied to understand the reaction adsorption mechanism(s) of Cr(VI) onto Fe_0.75_Cu_0.25_-BDC@Alg-MoO_3_/GO nanohybrid and the parameters of these four models are listed in Table 2. In Langmuir model, the behavior of adsorption mainly depends on a unimolecular chemical reversible process. It is obvious that the adsorption of Cr(VI) onto Fe_0.75_Cu_0.25_-BDC@Alg-MoO_3_/GO surface was most convenient to Langmuir model owing to the larger value of correlation coefficient (R^2^ 0.9934) as represented in Fig. 10b. This proves that the adsorption reaction was dependent on the formation of a homogeneous monolayer of loaded Cr(VI) onto Fe_0.75_Cu_0.25_-BDC@Alg-MoO_3_/GO nanohybrid in which all active sites possess equivalent attraction forces for adsorption of hexavalent chromium. The R_L_ value indicates whether the adsorption process is favorable or not. Herein, this was fraction values (0.05654–0.5451) to prove that the reaction between Cr(VI) and Fe_0.75_Cu_0.25_-BDC@Alg-MoO_3_/GO nanohybrid is a favorable adsorption process^56^.

**Table 2:** Adsorption isotherm models for Cr(VI) using 20 mg Fe_0.75_Cu_0.25_-BDC@Alg-MoO_3_/GO and shaking time = 30 min at pH 3.

Isotherm Model	Langmuir				Freundlich			D-R				Temkin		
Equation	$\frac{{C}_{e}}{{q}_{e}}=\left(\frac{1}{{q}_{max}}\right)b+\frac{{C}_{e}}{{q}_{max}}$ ${K}_{L}=1/(1+b{C}_{o})$				$ln{q}_{e}=ln{K}_{F}+\frac{1}{n}ln{C}_{e}$			$ln{q}_{e}=ln{q}_{s}+{(K}_{ad}{\varepsilon }^{2})$ $\varepsilon =RTln(1+\frac{1}{{C}_{e}})$				${q}_{e}=\frac{RT}{{b}_{T}}ln{a}_{T}+\frac{RT}{{b}_{T}}ln{C}_{e}$		
*Plot*	$${\raise0.7ex\hbox{${C_{e} }$} \!\mathord{\left/ {\vphantom {{C_{e} } {q_{e} }}}\right.\kern-\nulldelimiterspace} \!\lower0.7ex\hbox{${q_{e} }$}}$$ *vs. C*_*e*_				$$ln{q}_{e}$$ vs.$$ln{C}_{e}$$			$$ln{q}_{e}$$ vs.$${\varepsilon }^{2}$$				$${q}_{e}$$ vs.$$ln{C}_{e}$$		
Parameters	q_max_	b	R^2^	R_L_	n	K_F_	R^2^	q_s_	K_ad_	R^2^	E	a_T_	b_T_	R^2^
Units	mg g^-1^	Lmg^-1^	–	–	–	L mg^-1^	–	mg g^-1^	mol^2^J^-2^	–	kJ/mol	L/mg	J/mol	–
Cr(VI)	24.81	0.1669	**0.9934**	0.05654- 0.5451	2.182	4.154	**0.9862**	14.09	20.47	**0.7211**	0.2210	2.871	657.52	**0.9704**

Significant values are in bold.

Freundlich isotherm model postulated that the adsorption process could be established onto heterogeneous surface of the sorbent with unequal available active sites with the formation of multilayer adsorption^57^. The characterized R^2^ value was 0.9862 which is not as good as Langmuir model. This result states that the adsorption of Cr(VI) onto Fe_0.75_Cu_0.25_-BDC@Alg-MoO_3_/GO didn’t proceed on a heterogeneous surface. The n-value equal 2.182 to indicate a favorable adsorption process since n-value is greater than one. The adsorption mechanism by Dubinin-Radushkevich model depends on pore filling and postulates a multilayer character via physical process^58^. The characterized R^2^ value from the D-R model was 0.7211. Temkin model states that the reaction between the adsorbent and the adsorption depends on temperature with neglecting the concentration and also assumes that as the surface coverage increases the heat of adsorption declines linearly^59^. The R^2^ value of this model was identified as 0.9701. The R^2^ values D-R model and Temkin models referred to the inapplicability of these models to depict the mechanism for adsorption of Cr(VI) onto Fe_0.75_Cu_0.25_-BDC@Alg-MoO_3_/GO nanohybrid**.**

#### Impact of temperature and thermodynamics studies

**Figure 11:**
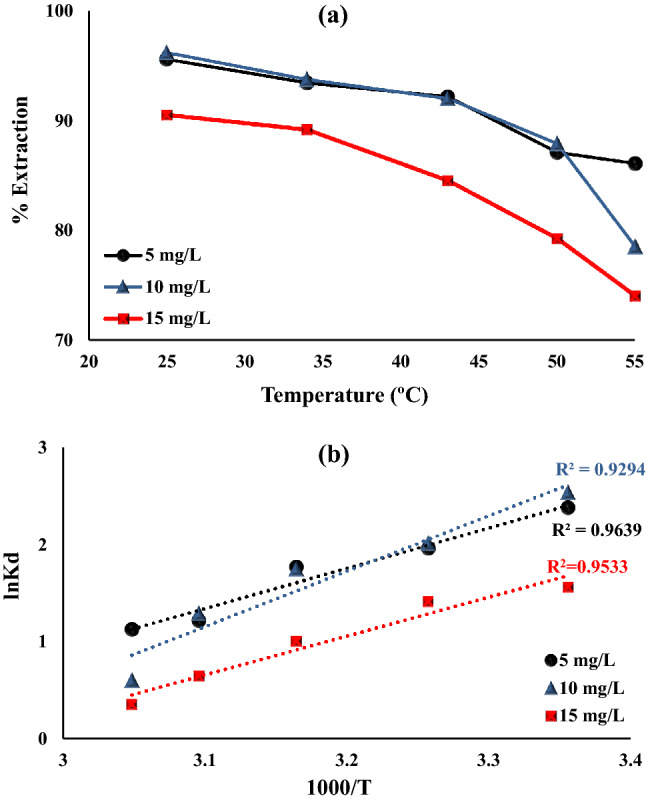
(**a**) Effect of temperature on Cr(VI) ions Fe_0.75_Cu_0.25_-BDC@Alg-MoO_3_/GO and (**b**) Van’t Hoff plot.

The adsorption behavior of Fe_0.75_Cu_0.25_-BDC@Alg-MoO_3_/GO nanohybrid towards hexavalent chromium was studied at various temperatures (293–328 K). This study showed that as the temperature increased from 293 to 328 K, the removal percentage decreased from 95.57% to 86.08%, 96.78% to 78.50% and 90.50% to 74.03% by using 5, 10 and 15 mg L^−1^ chromium ion concentrations, respectively. The decline in the adsorption at high temperatures may be attributed to the high mobility and solubility of hexavalent chromium in aqueous solution at higher temperatures ^60^. As illustrated in Fig. 11a, the plot between ln K_D_ versus 1000/T gives a slope (− ΔH^o^/R) and an intercept (ΔS^o^/R)from which the (ΔH^o^) and (ΔS^o^) values are evaluated using Van’t Hoff equation Fig. 11b. The calculated values of ΔG^o^, ΔH^o^ and ΔS^o^ from equations Eqs. 6–8 are compiled in Table 3. The negative values of Gibbs free energy displayed that the adsorption process Cr(VI) onto Fe_0.75_Cu_0.25_-BDC@Alg-MoO_3_/GO is spontaneous process. The values of enthalpy change are negative to confirm that the adsorption of Cr(VI) onto Fe_0.75_Cu_0.25_-BDC@Alg-MoO_3_/GO was an exothermic process. The values of entropy change were negative to prove that the adsorption process is an ordered process.

**Table 3:** Thermodynamic parameters.

Cr(VI)	Thermodynamics parameters		
	**ΔG**^**o**^	**ΔH**^**o**^	**ΔS**^**o**^
5 mg L^-1^	−5.89 −5.00 −4.65 −3.26 −3.07	−34.47	−93.65
10 mg L^−1^	−6.27 −5.13 −4.59 −3.45 −1.64	−47.43	−134.47
15 mg L^−1^	−3.86 −3.60 −2.63 −1.73 −0.96	−33.22	−95.41

6 $$\Delta G^\circ =-RTln{K}_{D}$$

7 $${K}_{D}=\frac{{q}_{e}}{{C}_{e}}$$

8 $$ln{K}_{D}=\frac{\Delta S^\circ }{R}-\frac{\Delta H^\circ }{RT}$$

Eventually, the evaluated parameters from the thermodynamic study prove that the adsorption behavior for adsorption of Cr(VI) onto Fe_0.75_Cu_0.25_-BDC@Alg-MoO_3_/GO nanohybrid was exothermic spontaneous ordered process.

#### Impact of ionic strength

**Figure 12:**
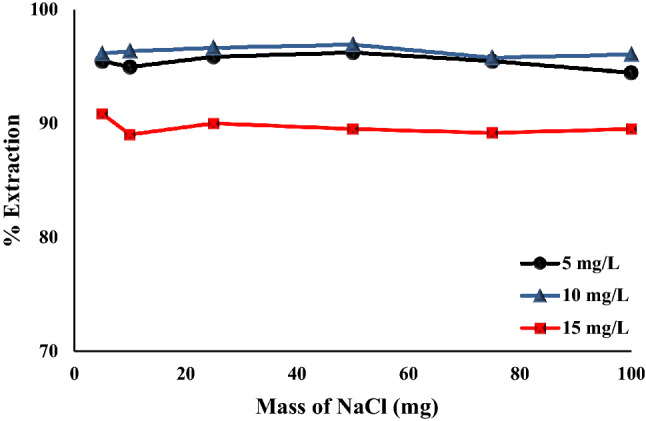
Effect of NaCl electrolyte on removal of Cr(VI) onto Fe_0.75_Cu_0.25_-BDC@Alg-MoO_3_/GO.

The presence of an electrolyte may affect the electrostatic attractions and binding mechanism between adsorbate (Cr(VI)) and adsorbent (Fe_0.75_Cu_0.25_-BDC@Alg-MoO_3_/GO). Figure 12 illustrates the effect of ionic strength by the addition of various doses of sodium chloride from 5 to 100 mg. It was found that the presence of NaCl slightly enhanced the removal percentage of Cr(VI) with percentages 95.44%, 96.01% and 90.85% by using 5, 10 and 15 mg L^−1^, respectively. This behavior may be based on the fact that under acidic conditions (pH 3) the surface of Fe_0.75_Cu_0.25_-BDC@Alg-MoO_3_/GO carries a positive charge and the presence of NaCl electrolyte increased the loaded positive charges on the surface to favor higher removal efficiency^61^.

### Reusability test of Fe_0.75_Cu_0.25_-BDC@Alg-MoO_3_/GO

**Figure 13:**
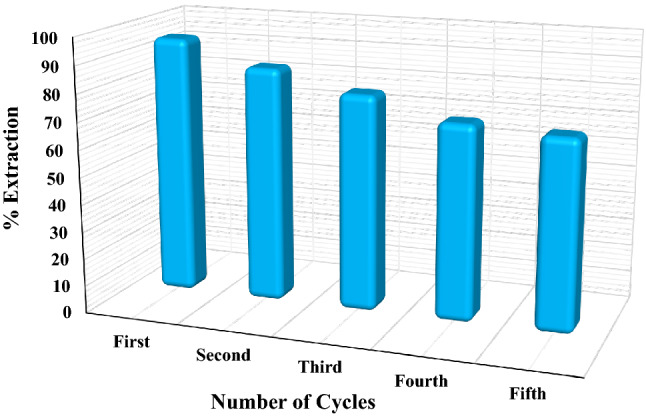
Recycling/reusability of Fe_0.75_Cu_0.25_-BDC@Alg-MoO_3_/GO.

The recycling capability of Fe_0.75_Cu_0.25_-BDC@Alg-MoO_3_/GO is a significant operation that must be studied to investigate the applicability of the prepared nanohybrid. Herein, the regeneration reagent was 0.05 mol L^−1^ HCl to activate and desorb the loaded Cr(VI) from the surface of Fe_0.75_Cu_0.25_-BDC@Alg-MoO_3_/GO nanohybrid. In this study, 100 mg of Fe_0.75_Cu_0.25_-BDC@Alg-MoO_3_/GO nanohybrid was mixed with 10 mL of Cr(VI) solution at pH 3. The loaded surface was then washed with HCl then DW several times and dried in an oven to be ready for further application after recycling operation. As illustrated in Fig. 13, five cycles were performed and the removal percentages of Cr(VI) by the recycled Fe_0.75_Cu_0.25_-BDC@Alg-MoO_3_/GO nanohybrid decreased from 95.21 to 70.04% after the fifth regeneration step. This decline may be attributed to the possible weight loss of Fe_0.75_Cu_0.25_-BDC@Alg-MoO_3_/GO nanohybrid. The outlined data in Fig. 13 prove the successful reusability of Fe_0.75_Cu_0.25_-BDC@Alg-MoO_3_/GO nanohybrid.

### Application of Fe_0.75_Cu_0.25_-BDC@Alg-MoO_3_/GO in Cr(VI) removal from real specimens

The validity of utilization of Fe_0.75_Cu_0.25_-BDC@Alg-MoO_3_/GO nanohybrid as efficient adsorbent material for the of hexavalent chromium ion from three different actual water specimens (tap, seawater, and wastewater) was studied by the batch technique. the water specimens were spiked with hexavalent chromium solution to obtain the three required concentrations of hexavalent chromium ion (5, 10 and 15 mg L^−1^). Then, the optimum conditions were used by adjustment of pH at 3 and using 70.0 mg of nanohybrid mass. The removal percentage values of hexavalent chromium from tap, seawater and wastewater were found to be more than of 98% as illustrated in Table 4. The outlined results emphasize that the prepared and investigated Fe_0.75_Cu_0.25_-BDC@Alg-MoO_3_/GO nanohybrid has been validated as an excellent regenerable adsorbent for removal of Cr(VI) from various water samples.

**Table 4:** Adsorptive removal of Cr(VI) by Fe_0.75_Cu_0.25_-BDC@Alg-MoO_3_/GO from real water samples at optimum conditions (Cr (VI) concentrations (5, 10 and 15 mgL^−1^) and 70 mg of adsorbent and pH 3).

Water sample	Percentage removal of Cr (VI)		
	**5 mgL**^**−1**^	**10 mgL**^**−1**^	**15 mgL**^**−1**^
Tap water	98.72	98.77	98.4
Sea water	98.62	98.50	98.20
Wastewater	98.83	98.01	98.85

## Conclusion

A novel nanohybrid (Fe_0.75_Cu_0.25_-BDC@Alg-MoO_3_/GO) has been prepared and investigated for remediation of water from hexavalent chromium. Inclusion of Fe_0.75_Cu_0.25_-BDC MOFs into Alg-MoO_3_/GO was accomplished by a simple and facile solvothermal process. Characterization of Fe_0.75_Cu_0.25_-BDC@Alg-MoO_3_/GO confirmed the successful preparation of with distributed particles homogeneously and uniformly at average particle size from 4.19 to 8.33 nm based on the TEM analysis and the point of zero charge of Fe_0.75_Cu_0.25_-BDC@Alg-MoO_3_/GO was found pH_Pzc_ = 3.8. Various parameters were studied to figure out the optimum conditions for extraction of Cr(VI) from aqueous solution (pH = 3 dosage, initial concentration of hexavalent chromium ion = 5, 10 and 15 mg L^−1^, shaking time = 5–10 min). The collected results from the kinetic study clarified that *pseudo*-second order model is the most convenient one to depict the adsorption behavior of Cr(VI) onto Fe_0.75_Cu_0.25_-BDC@Alg-MoO_3_/GO nanohybrid and the adsorption behavior was suggested to rely on a chemisorption mechanism. The impact of ionic strength by the presence of NaCl was found to slightly enhance the removal percentage of Cr(VI) due to the ability of Fe_0.75_Cu_0.25_-BDC@Alg-MoO_3_/GO to carry a more positive charge. The isotherm modeling study evaluated Langmuir model as the most valid model with a correlation coefficient R^2^ = 0.9934. The impact of temperature and characterized thermodynamic parameters revealed that the adsorption mechanism of Cr(VI) by Fe_0.75_Cu_0.25_-BDC@Alg-MoO_3_/GO was a spontaneous and exothermic process. The *pseudo*-second order model was proved as the most convenient model with R^2^ close to one (1.000, 0.9998 and 0.9993). Finally, the emerged Fe_0.75_Cu_0.25_-BDC@Alg-MoO_3_/GO could be classified and regarded as an effective adsorbent for removal of hexavalent chromium from real water specimens with percentages > 98%. Finally, the removal of Cr(VI) by Fe_0.75_Cu_0.25_-BDC@Alg-MoO_3_/GO nanocomposite was compared with previously reported adsorbents as compiled in in Table 5, indicating the superiority of the assembled and investigated Fe0.75Cu0.25-BDC@Alg-MoO3/GO nanocomposite in removal of Cr(VI) from aqueous solutions versus other previously reported adsorbent.

**Table 5:** Comparison of the maximum removal percentage of Cr(VI) by different adsorbents.

Contaminant	sorbent	Results (Maximum removal percentages)	Optimum pH	References
Cr(VI)	Silver impregnated groundnut husk carbon	≈ 97% of hexavalent chromium was removed within 5 h	3	^62^
	MGO-Trp	77.2%, 82.2% and 84.0% from tap, sea and waste water	2	^63^
	Cd–TIPA (Photocatalyst)	high catalytic efficiency 93%	–	^64^
	GO-CS@MOF [Zn(BDC)(DMF)]	≈ 92%	3	^65^
	ZrPO_4_-PPY nanocomposite	≈ 98.8% occurred in 80 min	2	^66^
	ZIF-8 Uio-66	≈ 85% ≈ 80	3	^67^
	ZIF-8@ABs UiO-66@Abs	≈ 60% ≈ 98%	6	^68^
	Fe_3_O_4_/ SiO_2_/CS-TETA	96.4%	2.5	^69^
	V_2_O_5_@Ch/Cu-TMA nanobiosorbent	96.61%, 96.95% and 95.72% from tap, sea and wastewater	3	^15^
	Fe_0.75_Cu_**0.25**_-BDC@Alg-MoO_3_-GO	> 98% from tap, sea and wastewater	3	This study

## Data Availability

The datasets used and/or analyzed during the current study are available from the corresponding author on reasonable request.
